# Injured patients who would benefit from expedited major trauma centre care: a consensus-based definition for the United Kingdom

**DOI:** 10.29045/14784726.2021.12.6.3.7

**Published:** 2021-12-01

**Authors:** Gordon Fuller, Samuel Keating, Janette Turner, Josh Miller, Chris Holt, Jason E. Smith, Fiona Lecky

**Affiliations:** University of Sheffield; University of Sheffield; University of Sheffield; West Midlands Ambulance Service; University of Sheffield; University Hospitals Plymouth NHS Trust; University of Sheffield

**Keywords:** expert consensus, injuries, major trauma, major trauma triage, reference standard, trauma centres

## Abstract

**Introduction:**

Despite the importance of treating the ‘right patient in the right place at the right time’, there is no gold standard for defining which patients should receive expedited major trauma centre (MTC) care. This study aimed to define a reference standard applicable to the United Kingdom (UK) National Health Service major trauma networks.

**Methods:**

A one-day facilitated roundtable expert consensus meeting was conducted at the University of Sheffield, UK, in September 2019. An expert panel of 17 clinicians was purposively sampled, representing all specialities relevant to major trauma management. A consultation process was subsequently held using focus groups with Public and Patient Involvement (PPI) representatives to review and confirm the proposed reference standard.

**Results:**

Four reference standard domains were identified, comprising: need for critical interventions; presence of significant individual anatomical injuries; burden of multiple minor injuries; and important patient attributes. Specific criteria were defined for each domain. PPI consultation confirmed all aspects of the reference standard. A coding algorithm to allow operationalisation in Trauma Audit and Research Network data was also formulated, allowing classification of any case submitted to their database for future research.

**Conclusions:**

This reference standard defines which patients would benefit from expedited MTC care. It could be used as the target for future pre-hospital injury triage tools, for setting best practice tariffs for trauma care reimbursement and to evaluate trauma network performance. Future research is recommended to compare patient characteristics, management and outcomes of the proposed definition with previously established reference standards.

## Introduction

Major trauma is an important public health problem in England, responsible for 3000 fatalities, 8000 severe disabilities, £0.4 billion of immediate treatment NHS costs and a £3.5 billion loss in economic output each year (Kehoe et al., 2015; National Audit Office, 2010). Major trauma is the leading cause of death in those aged under 40 and incidence is increasing in the ageing United Kingdom (UK) population (Kehoe et al., 2015). Improvements in the management of major trauma therefore have the potential to greatly improve both health and wealth (Cole et al., 2016; Lockey, 2018).

In 2012, major trauma care in England was reconfigured with the introduction of regional networks, aiming to concentrate seriously injured patients in specialist major trauma centres (MTCs) (Vondy & Willett, 2011). Non-MTC hospitals in England are classified either as trauma units (TUs), equivalent to American College of Surgeons Level III or IV trauma centre designation, or local emergency hospitals (LEHs), which do not routinely receive acute trauma patients. In accordance with NICE trauma guidelines, pre-hospital triage tools are used within trauma networks to identify which patients should be sent to MTCs (National Clinical Guideline Centre (UK), 2016). Bypass of non-MTC hospitals with less experience and expertise has been associated with improved patient outcomes (American College of Surgeons, 2006; Celso et al., 2006; Moran et al., 2018). However, research has suggested that existing tools are inaccurate and despite the importance of treating the ‘right person in the right place at the right time’ there is no clear ‘gold standard’ for defining which patients should be bypassed to an MTC (van Rein et al., 2017).

Injury Severity Score (ISS), an anatomical scoring system that measures the overall injury severity, has traditionally been used, with a threshold of ≥16 defining major trauma (Baker et al., 1974). However, as the ISS does not fully account for injury acuity, prognosis or futility, it has limitations as a measure for identifying patients who could benefit from expedited MTC care (Baxt & Upenieks, 1990; Newgard et al., 2008). Alternative, resource-based measures have been proposed reflecting the need for time-critical trauma-related clinical interventions (Lerner et al., 2014; Vassallo et al., 2020). However, these have been developed in the United States, or for major incidents, with questionable relevance to routine UK National Health Service (NHS) major trauma care.

This study therefore aimed to identify a reference standard applicable to the UK NHS major trauma networks for defining which patients should receive expedited MTC care. Such a definition would inform the development and validation of major trauma triage tools, help assess the performance of trauma networks and guide resource allocation.

## Methods

A one-day facilitated roundtable expert consensus meeting was conducted at the University of Sheffield, UK, on 4 September 2019 (Halcomb et al., 2008; Jones & Hunter, 1995). Seventeen clinicians were purposively sampled to form an expert clinical panel, ensuring that both MTCs and non-MTCs, and all specialties relevant to injury and major trauma management, were represented. Participants are detailed in Supplementary 1. The meeting was co-chaired by an independent University of Sheffield researcher (JT) and an established major trauma expert (JS).

Prior to the consensus meeting, literature searches were conducted to identify existing major trauma reference standards. The findings were summarised in preparatory materials and initial presentations to participants. The study’s aim of developing a ‘gold standard’ definition for injury that would benefit from expedited MTC care was emphasised, that is, defining which patients should be transported from the scene of injury with pre-alerting of the receiving MTC emergency department, and bypassing of a closer non-MTC if necessary, rather than severe injury per se. The meeting then proceeded in a structured format covering the scope, perspective, structure and components of the reference standard in repeated rounds of iterative discussions. Discussions were facilitated to ensure all issues were thoughtfully deliberated, incorporated diverse experience and views and produced the best possible decision (Halcomb et al., 2008; Jones & Hunter, 1995). Consensus was defined a priori as ‘finding a decision together that all members can feel comfortable with’ (Jones & Hunter, 1995, p. 376). Consensus was developed through group negotiation mediated by the meeting chairpersons.

Following the consensus meeting, separate focus groups were conducted with the Sheffield Emergency Care Forum and Birmingham Injuries Public and Patient Involvement (PPI) groups (Then et al., 2014). Focus groups began with a short presentation of the proposed reference standard and, following a series of familiarisation questions designed to build rapport, participants were asked to discuss their perspectives. A single moderator led all interviews (GF or JM) and a second researcher was present to take field notes (CH). Sessions continued until code saturation was reached (‘heard it all’). Discussions were audio recorded and later transcribed verbatim by a professional transcriptionist. Analysis followed the Framework Method, with familiarisation, thematic framework development, indexing, charting and mapping with interpretation (Gale et al., 2013).

PPI feedback was then circulated remotely to expert panel members via email, with any changes to the reference standard discussed remotely, before agreement was confirmed. Finally, to allow operationalisation of the reference standard in UK research a coding algorithm was developed with the English national trauma registry, the Trauma Audit and Research Network (TARN), facilitating classification of any case submitted to their database.

Study procedures, including sample size determination, followed recommended principles for best practice in developing consensus (Jones & Hunter, 1995). A study protocol was pre-specified. Ethical approval was provided by Yorkshire and The Humber – Bradford Leeds Research Ethics Committee (Reference: 19/YH/0197). All participants provided informed consent. The study was funded by the National Institute of Health Research Health Technology Assessment Programme (Grant: 17/16/04) as part of the larger Major Trauma Triage Study (MATTS) project.

## Results

Iterative rounds of facilitated discussions achieved consensus on four aspects of the reference standard for identifying patients who would benefit from expedited major trauma care: scope, perspective, structure and content.

In terms of scope, expert consensus was achieved that the reference standard should apply to patients presenting to NHS ambulance services, excluding major incidents and those involved in military action where treatment priorities may differ. Reference standard criteria were agreed to apply to patients aged over 16 years, conforming to organisation of NHS services for children and adults. Isolated burns were excluded due to the existence of separate NHS major burns networks, which may not overlap with major trauma networks. Cases with isolated hypoxic brain injury (e.g. drowning, non-judicial hangings) were judged ineligible as they usually lack concomitant physical injuries.

**Figure 1:**
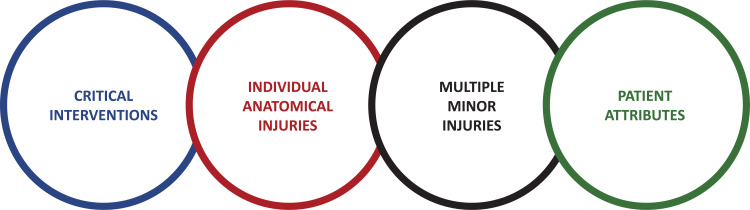
Figure 1. Schematic diagram of UK consensus definition of injury benefiting from major trauma centre care.

The reference standard perspective was agreed to characterise patients that would benefit from expedited MTC care, rather than injury severity singularly. There was also unison to develop a theoretical criterion standard initially, with later operationalisation for retrospective application and classification of cases to facilitate future research evaluating triage accuracy. A composite reference standard structure was confirmed comprising four domains of critical interventions, individual anatomical injuries, multiple minor injuries and patient attributes (Figure 1). This was designed to capture all injured patients with the potential to benefit from expedited MTC care, to allow operationalisation in trauma registry data and to minimise the disadvantages of existing single domain reference standards, for example ISS.

Individual criteria were defined by consensus within each domain, as detailed in Table 1. The Abbreviated Injury Scale (AIS) was chosen as the most comprehensive and validated system available to describe anatomical injuries (Greenspan et al., 1985). Each injury in the AIS 2005 (Update 2008) dictionary was examined individually for qualification as a condition benefiting from expedited MTC care in the NHS setting, rather than categorisation using an arbitrary AIS injury severity scoring rule, for example inclusion of all AIS 4+ injuries (Association for the Advancement of Automotive Medicine, 2016). Additional qualifiers were added to specific chest (presence of acute respiratory failure) and head (GCS thresholds) injuries to better capture clinical acuity. Finally, circumstances where expedited MTC care could not be beneficial (prolonged out-of-hospital traumatic cardiac arrest and catastrophic injuries) or would not be in a patient’s best interests (applicable advanced directive, advanced frailty) were confirmed.

**Table 1.** UK consensus definition of injury benefiting from major trauma centre care.

Domain	Critical interventions	Individual anatomical injuries	Multiple minor injuries	Patient attributes
**Rationale**	Injury requiring a time-critical trauma-related intervention only available, or more effectively delivered, in an MTC	Presence of significant individual anatomical injuries that would benefit from expedited MTC care	Presence of multiple injuries, non-significant in isolation, but cumulatively resulting in significant injury that would benefit from expedited MTC care	Exclusion of patients who do not want, or would not benefit from, expedited MTC care
**Criteria**	Resuscitation requiring any of: Emergency intubation Ventilation Blood product transfusion Indication for an emergency trauma-related surgical procedure (including ICP monitoring) Indication for emergency trauma-related interventional radiology Requirement for multidisciplinary critical care admission	Most AIS 4+ injuries, e.g. spinal injury with neurological deficit (unless can be satisfactorily managed in a non-MTC) Specific AIS <4 injuries: Significant de-gloving injury Open long bone fractures Amputations proximal to the fingers/toes Pelvic ring fractures (excluding osteoporotic fractures) Chest injuries with respiratory failure TBI causing GCS <9 if clinical frailty score <6 (residential home level care) TBI causing GCS 9–13 if clinical frailty score <6 and focal neurology or pupillary abnormalities Complex skull fractures: large, depressed areas, open with exposed brain, CSF leak	AIS injuries in 2+ body regions combining to ISS ≥16; total ISS ≥16 calculated by either: If presence of an AIS 4+ injury excluded from significant isolated injuries, then requires AIS 2+ injury in second body region If no AIS 4+ injury excluded from significant injuries, then include all AIS codes, except: Exclude all AIS = 1 Exclude all non-skeletal face AIS codes Exclude head region AIS codes in patients with clinical frailty score >=6 Exclude all ‘external and thermal’ and ‘other’ region codes Exclude ingestions and hypoxic brain injuries	Applicable advanced directives Clinical frailty score ≥7 (nursing home-level care) Un-survivable injuries, e.g. torso transection, massive brain destruction, decapitation Traumatic cardiac arrest >15 minutes transport from MTC

AIS: Abbreviated Injury Scale; CSF: cerebrospinal fluid; GCS: Glasgow Coma Scale; ICP: intracranial pressure monitoring; MTC: major trauma centre; TBI: traumatic brain injury.

Theoretical reference standard criteria were then operationalised by defining time intervals, identifying specific interventions, specifying clinical parameters and listing qualifying operations. These were then formulated according to data fields available in the TARN database (as detailed in Supplementary 2), together with the categorised AIS codes. A TARN coding algorithm was subsequently developed to allow retrospective application in research (available from TARN on request).

Focus groups with the Sheffield Emergency Care Forum and Birmingham Injury PPI groups included 12 and 15 participants, and lasted 63 and 94 minutes, respectively. Three important themes emerged, comprising reference standard structure, futility and patient choice. Participants were universally supportive of the four-domain reference standard structure. There was overwhelming agreement that ‘elderly frail patients should *not* be managed “aggressively”’. However, there were divergent opinions on whether age alone should be used as a criterion to determine whether a patient would benefit from expedited MTC care. Examples representing these conflicting viewpoints included ‘the Equity Act of 2012 makes it illegal to discriminate by age, and in any case 65 years old is too low!’ and ‘my parents are far too old to be going to [MTC name]’. In contrast, there was complete consensus that expedited MTC care would not be in the best interests of patients with advanced frailty. Finally, a minority of participants voiced strong opinions about patient choice in determining their desired location of care following injury, specifically referencing travelling distances and perceived quality of care in individual hospitals. Representative comments included: ‘Please remember that many older trauma patients will have carers who are also older and can’t travel far’, ‘an MTC probably provides better care for all levels of injury’ and ‘for me it depends which hospital they’re trying to take me to . . . I would never let them take me to [non-MTC name]’.

## Discussion

A reference standard to define which injured patients will benefit from expedited MTC care in the UK setting was developed through expert consensus and PPI consultation. Four domains were identified, comprising: need for critical interventions, presence of significant individual anatomical injuries, burden of multiple minor injuries and specific patient attributes. A coding algorithm to allow operationalisation in TARN data for future research was also created.

A fundamental principle in developing and choosing a valid reference standard for injured patients is specifying the required perspective and purpose. The most appropriate reference standard may vary according to the specific circumstance and it is therefore important to differentiate between foci of measurement of injury severity (often termed major trauma or polytrauma), risk of poor outcome and the potential to benefit from expedited MTC care. For the development of pre-hospital triage tools and assessing the performance of trauma networks, it is the need for expedited MTC care that is relevant, rather than the severity of injury or prognosis per se. For example, a patient with a fatal injury such as decapitation manifestly has major trauma but could not benefit from pre-hospital triage to an MTC.

Bypass of hospitals with less experience and expertise, with transportation to MTCs, has been associated with improved patient outcomes following injury (Moran et al., 2018). This may reflect improved resuscitation, initial management and investigation in MTCs; earlier delivery of interventions (surgical, interventional radiology, critical care or supportive) unavailable at a non-MTC; improved definitive care; and avoidance of the risks of adverse events during secondary transfers from a non-MTC to an MTC. However, there are several factors that could influence the appropriateness of expedited MTC care. Firstly, some patients with high ISS, but not requiring urgent resuscitation or specialist interventions, may be satisfactorily managed entirely in a non-MTC, or with a later planned secondary transfer if necessary. Aiming to bypass such patients could result in significant over-triage to MTCs, for no benefit in outcome (Lecky et al., 2017). Secondly, for patients with un-survivable injuries, or very severe comorbidities, outcomes may be fixed regardless of treatment, with bypass to MTCs being futile. Thirdly, in the context of advanced frailty and reduced performance status, the probability of improved outcome may be low compared to the burden of treatment. In these circumstances, local supportive or palliative care might be in a patient’s best interests, rather than distant MTC care. Finally, patients and families may prefer care closer to home in a local hospital and be willing to forego a better overall outcome to achieve this.

Traditionally injured patients have been classified according to an assessment of anatomical injuries using the AIS and ISS, with an ISS threshold ≥16 defining major trauma (Baker et al., 1974). While the ISS provides an overall estimate of injury severity, it has a number of potential limitations as a marker for benefit from expedited specialist MTC management (Palmer, 2007; Rutledge, 1996; Vassallo et al., 2020). The total number of contributing injuries to the ISS is limited to three, with equal importance to each body region, and an inability to account for multiple injuries to the same body region. Its calculation is confounded by the extent of radiological investigation performed. ISS has a non-linear relationship with mortality. Many different injury patterns, often with varying acuity and prognosis, can yield the same ISS. Furthermore, the ISS ≥16 threshold does not account for heterogeneity in intensity, urgency and complexity of treatments required for different injuries. Other anatomical reference standards, including the new injury severity score (NISS), have similar limitations (Osler et al., 1997).

Consequently, resource-based criteria have been proposed as an alternative to anatomical measures to define major trauma. A number of consensus-based definitions have been formulated focusing on identifying patients who need specialised trauma interventions that are unavailable, or less effectively delivered, in non-MTCs (Lerner et al., 2014; Vassallo et al., 2020). However, purely resource-based definitions of major trauma also have limitations for reflecting benefit from expedited MTC care. Retrospective classification is subject to variability in medical decision-making and hospital expertise, and may not reflect the clinical indication for an intervention. For example, it has been demonstrated that splenic injuries which would usually be managed conservatively in most MTCs may undergo inappropriate emergency splenectomy if managed in non-MTCs (Green et al., 2020; Yiannoullou et al., 2017). There may also be large inherent resource needs despite mild anatomic injury in those suffering a concomitant acute medical event. Improved outcomes secondary to early MTC management over and above emergency interventions, such as improved resuscitation, supportive care or investigations, are additionally not accounted for.

Prognostic reference standards can also be used to define severity, using combinations of mechanistic, anatomical, physiological and demographic variables to predict mortality after injury (de Munter et al., 2017; Sewalt et al., 2020). A probability threshold could then be defined above which the risk of adverse outcome is judged to justify MTC care. While numerous prediction models have been developed, they have largely demonstrated suboptimal performance (de Munter et al., 2017; Sewalt et al., 2020). Further issues with this approach include justifying an arbitrary cut-off point to represent the need for MTC care, a focus on mortality rather than morbidity and not accounting for whether MTC care would change outcome. The advantages and disadvantages of existing reference standards for injured patients are summarised in Table 2.

**Table 2.** Existing reference standards for major trauma.

Category	Anatomical	Resource	Prognosis	Composite
**Examples**	Injury severity score New injury severity score Abbreviated Injury Scale Swiss highly specialised medicine regulations	US consensus definitions Vassallo definition	Revised trauma score TRISS TARN prediction model New revised trauma score MGAP Emergency Trauma Score A Severity Characterisation of Trauma (ASCOT) ICD-based injury severity score (ICISS) Trauma Mortality Prediction Model Polytrauma score Acute Trauma Index CRAMS Pre-hospital Index (PHI) Triage Revised Trauma Score (T-RTS) Physiologic Severity Score (PSS) Mechanism, Glasgow Coma Scale, Age and Arterial Pressure (MGAP) Modified Rapid Emergency Medicine Score (mREMS) Kampala Trauma Score (KTS) Berlin polytrauma criteria	Current MATTS reference standard German trauma society criteria Ad hoc composite measures, e.g. combination of ISS, intensive care unit admission and any non-orthopaedic surgery within 24 hours
**Advantages of defining need for expedited MTC care**	*Theoretical*: Simple Objective *Operational*: Readily available for research purposes from trauma registries	*Theoretical*: Captures patients with low ISS who need urgent trauma interventions Reflects critical determinants of benefits of MTC care *Operational*: Readily available for research purposes from trauma registries	*Theoretical*: Objective Presents mortality risk range/threshold *Operational*: Information for calculation available from trauma registries	*Theoretical*: Minimises the disadvantages of single domain reference standards *Operational*: Information for calculation available from trauma registries
**Disadvantages of defining need for expedited MTC care**	*Theoretical*: Some apparently less severe injuries benefit from urgent MTC interventions Modifiers of injury severity excluded, e.g. comorbidities Injury severity not fully captured, e.g. GCS score in TBI Futility excluded (except AIS 6 un-survivable injuries) Patient values excluded *Operational*: Influenced by degree of investigation	*Theoretical*: Some serious injuries benefiting from MTC care may not need resource-based interventions Patient values excluded *Operational*: Subject to variability in medical decision-making and hospital expertise Potential confounding effect from concomitant acute medical events	*Theoretical*: Significance of individual injuries excluded Resource use excluded Futility excluded Patient values excluded *Operational*: Often complex Generally poor predictive performance Often unvalidated	*Theoretical*: Might compound the disadvantages of single domain reference standard *Operational*: Increased complexity

The inclusion of UK major trauma experts should ensure this reference standard is valid throughout the NHS. The proposed composite structure aims to maximise the advantages, and minimise the drawbacks, of single-domain anatomical, resource-based or prognostic reference standards. There is consequently substantial overlap with the US consensus definition of major trauma and ISS (Baker et al., 1974; Lerner et al., 2014); but with the important differences of inclusion of significant individual injuries, finessing of the traditional ISS ≥16 threshold, more nuanced chest and traumatic brain injury criteria and consideration of clinical frailty scores. Specific individual injuries that could benefit from bypass to MTCs are captured, such as chest injuries with respiratory failure, or open fractures. The traumatic brain injury criteria will include any patient where emergency neurosurgery or intracranial pressure monitoring is indicated but allows for the fact that head injury has a poor prognosis with advanced age, increasing frailty and multi-morbidity (Gardner et al., 2018).

This study has several strengths, including a comprehensive sample of MTC and non-MTC experts, PPI input and conformity with consensus study guidelines (Halcomb et al., 2008; Jones & Hunter, 1995). The consensus approach allowed fuller deliberation of the decision problem, compared to other decision-making approaches such as executive decisions or majority rule. Roundtable methodology is better suited to complex, multi-dimension constructs than other consensus methodology such as Delphi process or nominal group technique, which are designed to answer narrow, single issue questions (Halcomb et al., 2008; Jones & Hunter, 1995). However, there are some limitations. The geographical spread of included experts was relatively restricted, and although generalisable to the UK, external validity of the proposed reference standard to other settings is less likely, due to differences in trauma system configuration, health service organisation, patient values and accepted clinical standards of care. Moreover, it is possible that individual views within the expert panel may not be fully consistent with evidence-based practice or could be unrepresentative of general opinion within each clinical specialty. Lastly, while the final reference standard was agreed by the whole expert panel, when highly specialist topics were discussed the specific clinical expert may have a dominant input.

In conclusion, we present an expert consensus criteria (or reference standard) to identify injured patients who would benefit from expedited major trauma care. This reference standard could be used as the target for future pre-hospital injury triage tools, for setting best practice tariffs for trauma care reimbursement and for evaluating trauma network performance. Future research is recommended to compare patient characteristics, management and outcomes between the proposed definition and existing reference standards.

## Acknowledgements

We would like to acknowledge members of the MATTS reference standard consensus panel for their help and expertise: Jason Smith, Tim Coats, Fiona Lecky, Stuart Reid, Kay Stenton, Bill Bailey, Adel Helmy, Stuart Harrison, Jonathan Edwards, Jeff Garner, Mike Dennison, Ian Carmichael, Edward Mills, Johnathan May, Jonathan Jones, Gary Mills and Johno Breeze. We are also very grateful to the Sheffield Emergency Care Forum and Birmingham Injury PPI groups for their help and support.

## Author contributions

GF conceived the study. GF and SK managed the study. JS and JT co-chaired the consensus meeting. The MATTS reference standard consensus panel developed the definition of injury benefiting from expedited MTC care. JM and CH performed the PPI focus groups. All authors made substantial contributions to the design, data processing and interpretation. All authors had full access to all of the data in the study and can take responsibility for the integrity of the data and the accuracy of the data analysis. JT acts as the guarantor for this article.

## Conflict of interest

None declared.

## Ethics

Ethical approval was provided by Yorkshire and The Humber – Bradford Leeds Research Ethics Committee (Reference: 19/YH/0197).

## Funding

None.
